# A novel null allele of *C. elegans* gene *ceh-14*

**DOI:** 10.17912/g434-3d85

**Published:** 2018-10-18

**Authors:** Emily Bayer, Oliver Hobert

**Affiliations:** 1 Department of Biological Sciences, Columbia University, New York, NY, USA; 2 Howard Hughes Medical Institute

**Figure 1:**
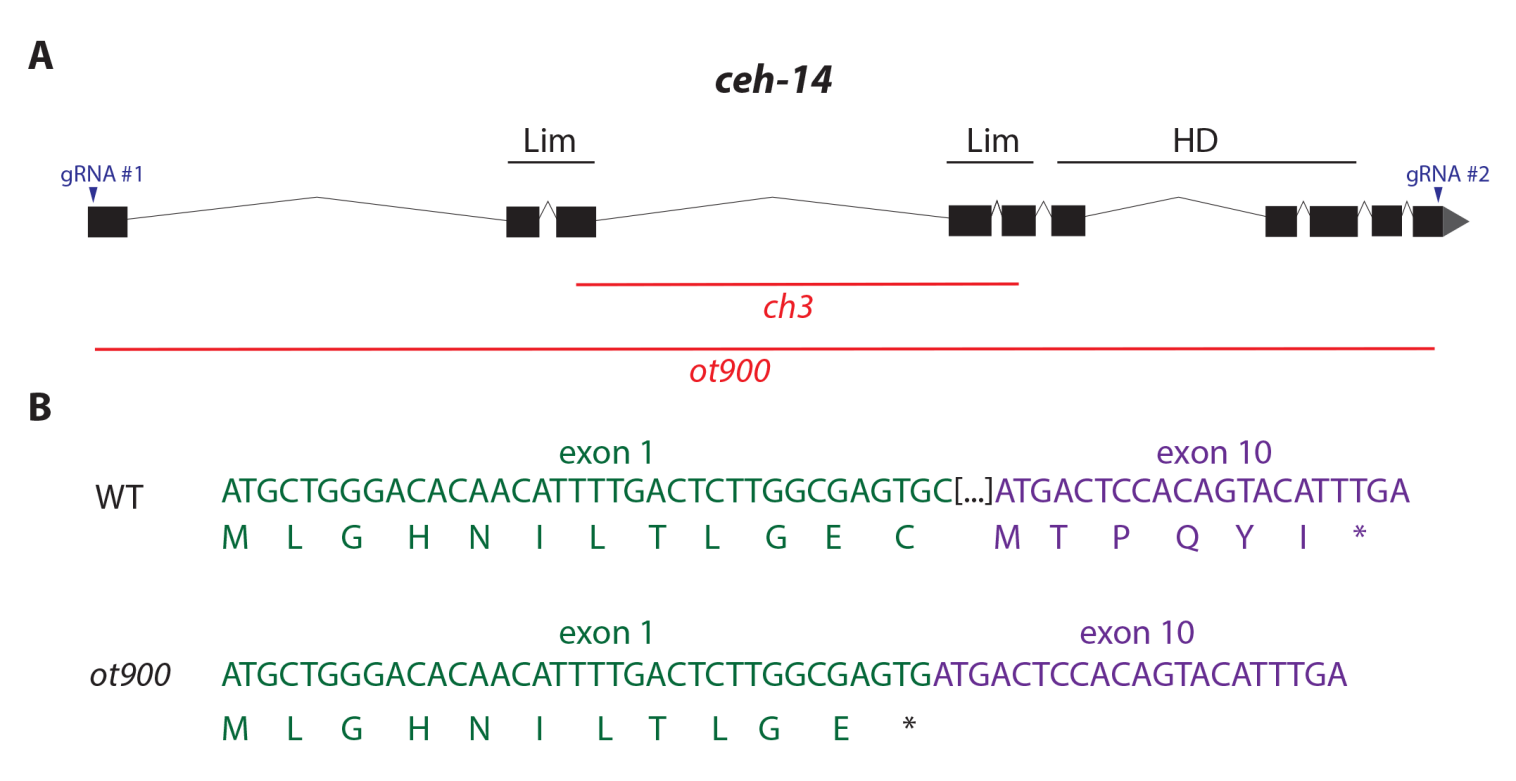
*ot900* is a novel null allele of *ceh-14*. We used gRNAs located in the first and last exons of *ceh-14* to generate a large deletion allele spanning the *ceh-14* ORF (A). The remaining 35bp in exon 1 are capable of generating an 11aa peptide before a deletion-induced frame-shift results in a premature stop (B).

## Description

We have generated a novel null allele, *ot900*, of the *C. elegans* LIM homeodomain gene *ceh-14*. All existing deletion alleles of *ceh-14* were generated using a Tc1 transposon insertion, one of which, *ch3*, is a putative null based on the resulting frame-shift and a reduction in detectable mRNA and protein levels (Cassata et al. 2000). However, in our hands we have noticed a propensity of the *ch3* allele to revert to wild-type after several generations, perhaps due to a more complex rearrangement of the locus caused by the Tc1 transposon than previously appreciated.

To avoid potential complexities of working with the *ch3* allele, we used CRISPR/Cas9 to generate a new null allele of *ceh-14*. The *ot900* allele was isolated with gRNAs targeted to the first and last exons of *ceh-14* (gRNA #1: CTTGGCGAGTGCGATGAGC, gRNA #2: GTACTGTGGAGTCATGTGT; see Fig. 1), and then PCR screening for resulting large deletion alleles. Our primers for PCR genotyping of the deletion are 5’-CTCAACTAAATCGTCAGAATCGTC-3’ and 5’-GAGATGTATGAAACGAGCGAGCG-3’ which generate a 620bp product in the context of the *ot900* deletion. The *ceh-14* locus in the *ot900* allele is only capable of generating an 11aa peptide due to the remaining bases in the first exon, before reaching a premature stop codon (Fig 1B).

Previous studies have implicated *ceh-14* in differentiation and function of the phasmid neurons, but have shown incomplete penetrance and variability in phenotypes resulting from the *ceh-14 (ch3)* allele (Kagoshima et al. 2013, Serrano-Saiz et al. 2015). We used the *srg-13* marker of PHA identity (which was expressed in 20/20 wild-type animals) to assess whether phasmid neuron differentiation is more severely affected in *ot900* than *ch3,* and found that this marker was indeed lost in 100% of animals (expressed in 0/15 animals), despite not finding an appreciable effect in *ceh-14 (ch3)* (expressed in 15/15 animals)*.* This suggests that some of the variability in phenotype may be the result of the *ch3* allele itself, rather than a property of CEH-14 function.

## Reagents

OH15422 *ceh-14 (ot900)* X. Will be available at CGC.
